# Dislocation reduction of InAs nanofins prepared on Si substrate using metal-organic vapor-phase epitaxy

**DOI:** 10.1186/1556-276X-7-642

**Published:** 2012-11-23

**Authors:** Chao-Wei Hsu, Yung-Feng Chen, Yan-Kuin Su

**Affiliations:** 1 Department of Electrical Engineering & Advanced Optoelectronic Technology Center, Institute of Microelectronics, National Cheng Kung University, 1 University Rd, Tainan City, 701, Taiwan

**Keywords:** Threading dislocations, Metal-organic vapor-phase epitaxy, InAs, Nanofins

## Abstract

InAs nanofins were prepared on a nanopatterned Si (001) substrate by metal-organic vapor-phase epitaxy. The threading dislocations, stacked on the lowest-energy-facet plane {111}, move along the SiO_2_ walls, resulting in a dislocation reduction, as confirmed by transmission electron microscopy. The dislocations were trapped within a thin InAs epilayer. The obtained 90-nm-wide InAs nanofins with an almost etching-pit-free surface do not require complex intermediate-layer epitaxial growth processes and large thickness typically required for conventional epitaxial growth.

## Background

The semiconductor industry has followed Moore's law for over 30 years with gate dimensions of transistors approaching the nanometer scale (<100 nm) [1]. The nanofin channels that are partly wrapped around these gates offer more effective control than that in traditional planar devices [2]. InAs is a direct-bandgap transition semiconductor material with high electron mobility [1-4]. InAs on a Si substrate is expected to allow side-by-side integration of InAs optoelectronics with conventional Si-based complementary metal oxide semiconductor products using monolithic epitaxial technology [1-8]. Unfortunately, the hetero-epitaxy of InAs nanofins deposited on Si presents challenges, including the dislocations on the nanofin surface and positioning of the nanofin. These dislocations are generated due to an 11.6% lattice constant mismatch (*a*_Si_ = 5.43 Å, *a*_InAs_ = 6.06 Å), thermal expansion coefficient mismatch, and polarity mismatch [6-8]. For a zinc-blende structure deposited on a diamond structure, the dislocations generated near the InAs/Si interface, such as mistfit dislocations, threading dislocations, and antiphase domain boundaries, are annihilated or multiplied during the epitaxial process [6-19]. A previous report indicated that a threading dislocation density above 10^7^ cm^−2^ for a 900-nm-thick epilayer decreased to 10^6^ cm^−2^ due to the self-annihilation of dislocations when the film thickness was increased to more than 4 μm for a III-V epilayer deposited on a Si substrate [8,18]. A III-V epilayer on a miscut Si (001) substrate has fewer threading dislocations and antiphase domain boundaries than those of an epilayer on an untilted Si (001) substrate [6,17]. Previous studies have attempted to reduce the dislocation density for a III-V epilayer deposited on a Si (001) substrate using methods such as thermal cycle annealing and the utilization of short-period strained intermediate-layer superlattices [6-10]. During thermal cycle annealing treatment, an epilayer is subjected to large temperature oscillations and thus periodically switches between the compressed and tensile states, reversing the motion of the dislocations. However, thermal cycle annealing processes are time-consuming. Strained intermediate-layer superlattices effectively decrease the threading dislocation density by generating additional stress, but their insertion leads to poor reproduction. In previous reports, the density of dislocations in an epilayer deposited on a planar Si substrate could be decreased by about two orders of magnitude by mixed thermal cycle annealing steps and strained intermediate-layer superlattice epitaxial growth processes [6-10]. An alternative technology for effectively reducing dislocation density is a kind of selective epitaxial growth on patterned Si substrate, which has the capability to produce a low-threading-dislocation surface using a simple epitaxial growth process. Yamaguchi et al*.* reported a model for the etching-pit density that could be reduced as the pattern trench width decreases [19].

In this work, InAs nanofins were deposited on nanopatterned Si (001) with SiO_2_ as sidewalls. As confirmed by transmission electron microscopy (TEM), the high-aspect-ratio (aspect ratio = trench height / trench width) SiO_2_ sidewalls produce an almost etching-pit-free InAs surface. The 90-nm-wide InAs nanofins with almost etching-pit-free surfaces represent a breakthrough for a III-V material for use in the advanced Si-based semiconductor industry.

## Methods

InAs nanofin growth was performed in an Aixtron (Herzogenrath, Germany) metal-organic vapor-phase epitaxy reactor using H_2_ as the carrier gas, with a total flow of 6,000 sccm. A nanoscale shallow trench isolation pattern, with the narrowest width below 100 nm, and a miscut of 6° towards the $\left[1\overset{―}{1}0\right]$ direction on p-type Si (001) wafers were adopted in this work. For patterned substrate fabrication, a 235-nm-thick SiO_2_ film was formed by thermal oxidation on a Si (001) substrate. The SiO_2_ mask patterns were produced using 193-nm immersion lithography and reactive ion beam etching on a (001)-oriented Si substrate. The nanopatterned Si substrates were then cleaned in a 1:1:7 solution of reagent-grade HCl/H_2_O_2_/H_2_O for 5 min prior to 1 vol.% HF for 1 min. After a thermal desorption procedure, low-temperature InAs nucleation layers were selectively grown on a patterned Si (001) substrate at 623 K and a pressure of 37.5 Torr. High-temperature InAs buffer layers were then selectively grown at 823 K and a pressure of 75.0 Torr. The surface morphology was studied using field-emission scanning electron microscopy (SEM). High-resolution cross-sectional TEM images were obtained using an FEI Tecnai F20 microscope (FEI, Hillsboro, OR, USA) operated at 200 kV.

## Results and discussion

**Figure 1:**
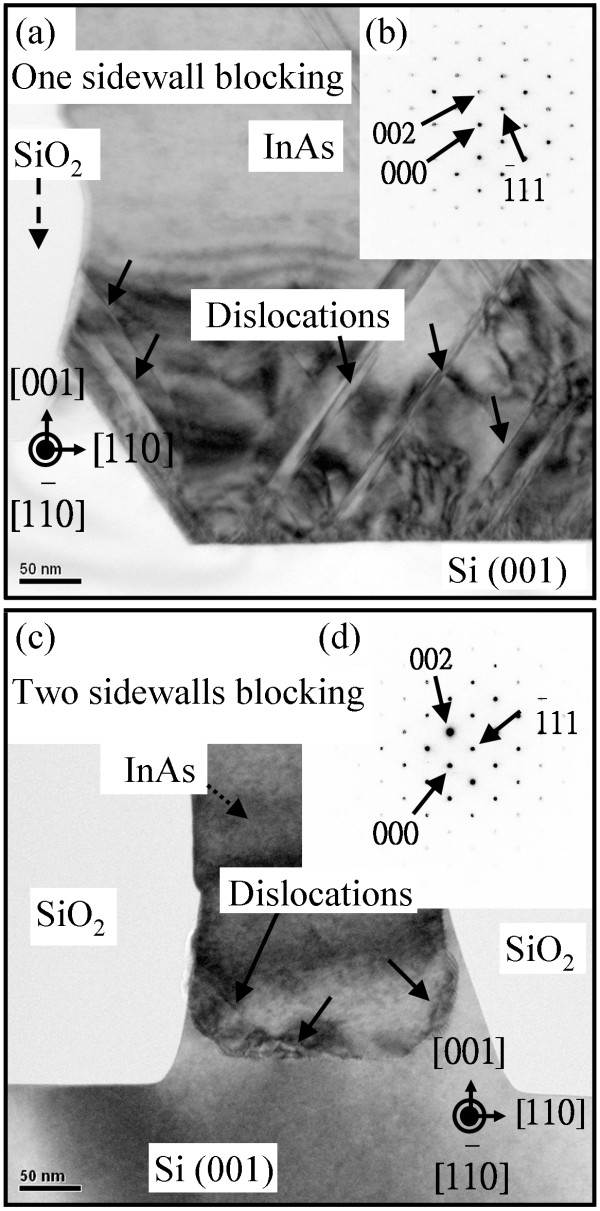
**One-sidewall and two-sidewall blocking. (a)** Cross-sectional TEM image and **(b)** diffraction pattern taken near the trench top region for InAs deposited on a one-sidewall-patterned Si (001) substrate. **(c)** Cross-sectional TEM image and **(d)** diffraction pattern taken near the trench top region for InAs deposited on a 200-nm-wide patterned Si (001) substrate with an aspect ratio of 1.2. The dislocations are marked with arrows.

Figure 1a,b shows a cross-sectional TEM image and the diffraction pattern of InAs deposited on a one-SiO_2_-sidewall-patterned Si (001) substrate, respectively. The lattice constant of the InAs epilayer is about 5.96 Å. The arrows indicate the threading dislocations. A large number of dislocations were generated at the InAs/Si interface; the dislocation density is above 10^9^ cm^−2^. In Figure 1a, the dislocations settle at an incline angle to the Si (001) surface, and some dislocations are blocked by the one-SiO_2_-sidewall pattern. However, this one-SiO_2_-sidewall pattern cannot effectively prevent the dislocations from prorogating upward. Figure 1c shows a TEM image of InAs grown on a 200-nm-wide patterned Si (001) substrate with an aspect ratio of 1.2. The dislocations are blocked within the initial epilayer with the two-SiO_2_-sidewall-patterned Si substrate. The diffraction pattern of the InAs epilayer on a 200-nm-wide trench-patterned Si (001) substrate is shown in Figure 1d. The lattice constant is about 6.03 Å, which is similar to that of natural InAs (6.06 Å). The *d*_002_ value (*d*_002_ = 3.029 Å) of InAs on nanopatterned Si substrate is similar to that of normal InAs (*d*_002_ = 3.030 Å). A low-residual-strain InAs epilayer was obtained using the nanopatterned Si substrate. In addition, the diffraction pattern shows no twin spots, confirming a lack of twin defects. According to the diffraction pattern, the epitaxial direction of InAs is along the [001] direction. The InAs has a coherent direction with the (001)-oriented Si substrate.

**Figure 2:**
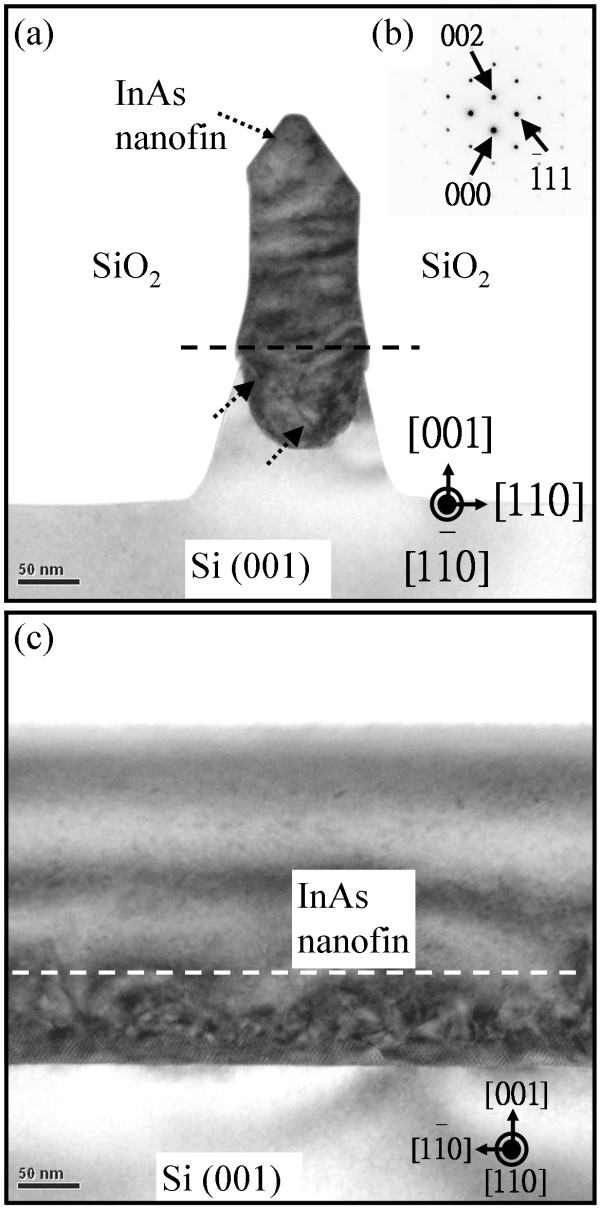
**InAs deposited on a 90-nm-wide-patterned Si (001) substrate with an aspect ratio of 2.5. (a)** Cross-sectional TEM image, **(b)** selected-area electron diffraction pattern taken near the trench top region, and **(c)** cross-sectional TEM cleft images along the $\left[1\overset{―}{1}0\right]$ direction of the InAs nanofins for InAs deposited on a 90-nm-wide-patterned Si (001) substrate with an aspect ratio of 2.5. The dislocation interruption is marked with a dashed line, and the dislocations are marked with arrows.

Figure 2a,b shows a TEM image and the diffraction pattern of InAs grown on a 90-nm-wide trench-patterned Si (001) substrate with an aspect ratio of 2.5, respectively. The $\left[1\overset{―}{1}0\right]$ direction is the short axis of the InAs nanofins. The lattice constant of the InAs epilayer is about 6.04 Å. Trenches with an aspect ratio of 2.5 are effective in stopping the extension of dislocations. The dislocations generated in the zinc-blende (InAs) and diamond structure (Si) interface include misfit and threading dislocations. Misfit dislocations are generated from the InAs/Si interface and are parallel to the growth plane [15]. Theoretical models have proposed the possibility of strain relief by decreasing the initiating epitaxial areas to the nanoscale regime [19-24]. The effect of strain transfer and dilution for finite dimensional nanopatterned substrate is considered as the linear dimension of individual nanoscale areas is reduced to the point where the stress is completely relieved before sufficient energy is accumulated to create a dislocation. The almost dislocation-free epilayer is possibly demonstrated when the epilayer was deposited on a nanoscale epitaxial area. However, we observed the dislocations, as shown in Figure 2a. The stain energy in the trench middle region is higher than the trench edge region [20-23]. The dislocations are generated in the trench middle region due to strain relaxation. Then, the dislocations extended to the trench edge region. Figure 2c shows a cross-sectional TEM image along the $\left[1\overset{―}{1}0\right]$ direction of the sample in Figure 2a. The $\left[1\overset{―}{1}0\right]$ direction is the long axis of the InAs nanofins. The dislocations are trapped within the initial epilayer. It is anticipated that the upward propagation dislocations are along the $\left[1\overset{―}{1}0\right]$ direction (the short axis of the nanofins). Park et al*.* reported misfit segments mostly along the $\left[1\overset{―}{1}0\right]$ direction (the short axis of the nanofins) for the epilayer deposited on submicron-patterned Si (001) substrate [25]. Misfit segments can lie along the $\left[1\overset{―}{1}0\right]$ direction in the (001) growth plane, while threading segments rise up on the {111} plane in the same direction [6,25]. Threading dislocations on the {111} plane are a result of the planes' lower facet surface energy [13,14]. Threading dislocations make a 45° angle with the underlying Si (001) substrate. The threading dislocations can be stopped by the SiO_2_ sidewalls on the Si (001) substrate. It is anticipated that almost all the dislocations will be blocked when the aspect ratio is greater than tan45° (aspect ratio > 1 [width × tan45°]). For InAs deposited on a 90-nm-wide trench-patterned Si (001) substrate, the dislocations can be trapped at a thickness below approximately 90 nm. As shown in Figure 2c, a displacement-type moiré pattern of the InAs initial deposition layer, which reflects the usual characteristic of a regular misfit direction, is observed at the InAs/Si interface. The InAs deposited on the nanopatterned Si (001) is almost dislocation-free on the top epilayer region. The pattern aspect ratio of 2.5 is good enough to block the dislocations from propagating upward.

**Figure 3:**
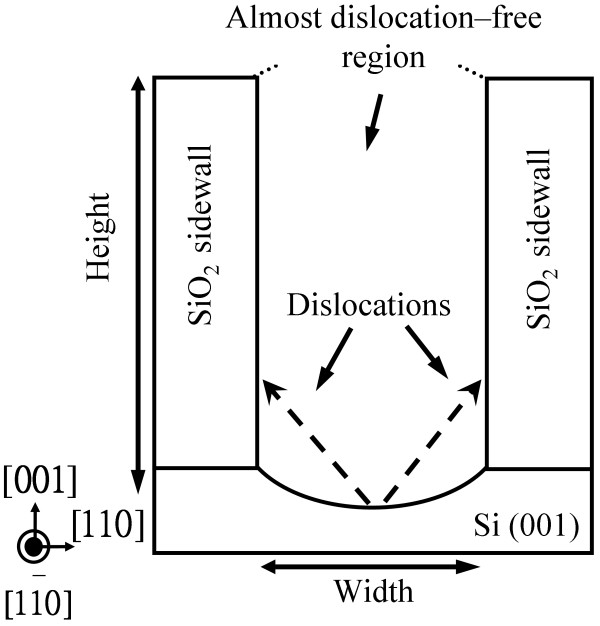
Principle of the dislocation-trapping mechanism and the almost dislocation-free top epilayer region.

A schematic diagram of the dislocation-trapping mechanism is shown in Figure 3. Compared with the rectangular-planar-shaped trench bottom, the concave-shaped trench bottom helps the formation of double steps, resulting in the suppression of antiphase domain boundaries [12]. The concave-shaped bottom, related to the double-step formation energetics on the substrate surface, facilitates surface step creation. Compared with InAs on a Si (111) substrate for a large number of vertical upward dislocations, the InAs on a Si (001) substrate can avoid the vertical upward dislocations. Compared to a one-SiO_2_-sidewall-patterned Si substrate, a high-aspect-ratio nanopatterned Si substrate with two SiO_2_ sidewalls can decrease the dislocation density from about 10^9^ cm^−2^ to almost 0.

**Figure 4:**
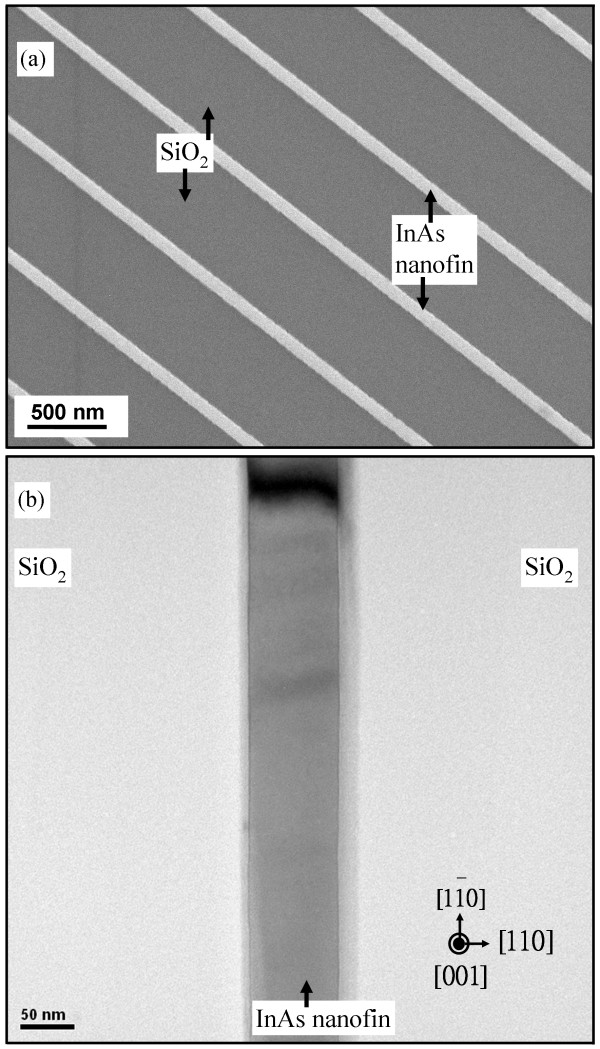
**The 90-nm-wide InAs nanofins on nanopatterned Si substrate.** SEM **(a)** and high-resolution TEM **(b)** images of the 90-nm-wide InAs nanofins on nanopatterned Si substrate with SiO_2_ as sidewalls.

In previous reports, the etching-pit density of the epilayer deposited on a planar Si substrate was reduced by more than one order of magnitude to about 1.2 × 10^6^ cm^−2^ using thermal cycle annealing and strained intermediate-layer superlattices [9,10]. Another study reported an etching-pit density of 2 × 10^6^ cm^−2^ for epilayer deposited on a planar Si substrate using GeSi intermediate layers [26]. Another study found an etching-pit density for an epilayer deposited on submicron domain-patterned and planar Si substrates of 6 × 10^5^ and 4 × 10^7^ cm^−2^, respectively [27]. The molten KOH etching was used to examine the dislocation behavior on the epilayer surface [15,28,29]. In this study, the samples were immersed in molten KOH solution at 633 K for 2 min, and then, the standard cleaning steps were used to clean the sample surface. Figure 4a shows a SEM image of an InAs nanofin surface. The InAs nanofin was selectively deposited in individual open Si surfaces bounded by the SiO_2_ mask. The length of the nanofins is above 4.5 μm. A high-resolution TEM image of the InAs nanofin surface is shown in Figure 4b. The two images indicate an almost etching-pit-free InAs nanofin surface. Corresponding to Figure 2a,c, the dislocations generated from the InAs/Si interface were trapped, and thus, an almost etching-pit-free surface was created, as shown in Figure 4a,b. Gao et al*.* reported vertical InP nanowires epitaxially grown on Si (111) by metal-organic vapor-phase epitaxy (MOVPE) [30]. Liquid indium droplets were formed *in situ* and used to catalyze deposition. Recently, Miao et al*.* demonstrated a catalyst-free InP nanowire growth on Si (001) by MOVPE [31]. The density and size of the nanowires can be controlled, but the positions of the nanowires are difficult to define. In this study, we used the immersion lithography technique to define the nanoscale epitaxial position. InAs nanofins (a nanostructure similar to nanowires) on a nanopatterned Si (001) substrate are suitable for the advanced commercial integrated circuit industry. InAs deposited on a nanopatterned Si substrate is a viable option for a good template for III-V epilayer integrated circuits for use with Si-based systems.

## Conclusions

The 90-nm-wide InAs nanofins were selectively deposited on a nanopatterned Si (001) substrate with SiO_2_ as sidewalls. As confirmed by TEM, the InAs deposited on the nanopatterned Si (001) shows an almost dislocation-free top epilayer region. The dislocations, originating from the InAs/Si interface, extended along the {111} facet plane and were terminated by two SiO_2_ sidewalls. When the aspect ratio is greater than 1, the dislocations can be trapped within an initial thickness (thickness < 100 nm). The 90-nm-wide InAs nanofin surface is almost etching-pit-free. InAs nanofins, with a width of about 90 nm, have the potential to be incorporated into the advanced Si-based complementary metal oxide semiconductor technology platform, allowing InAs material to be integrated with Si (001) substrates.

## Competing interests

The authors declare that they have no competing interests.

## Authors' contributions

C-WH participated in the material preparation and data analysis and drafted the manuscript. Y-KS conceived of and co-wrote the paper. Y-FC participated in the sample characterization. All authors read and approved the final manuscript.

## Authors' information

C-WH and Y-FC are postdoctoral researchers in the Institute of Microelectronics and the Department of Electronic Engineering, National Cheng Kung University, Tainan, Taiwan. Y-KS is currently a professor in the Institute of Microelectronics and the Department of Electrical Engineering, National Cheng Kung University, Tainan, Taiwan. Y-KS has authored or co-authored over 200 papers in the fields of thin-film materials, compound semiconductors, integrated optics, and optoelectronic devices.

## References

[B1] Chau R Datta S Doczy M Doyle B Jin B Kavalieros J Majumdar A Metz M Radosavljevic M Benchmarking nanotechnology for high-performance and low-power logic transistor applications IEEE Trans Nanotechnol 2005 4 153 158 10.1109/TNANO.2004.842073

[B2] Blekker K Münstermann B Matiss A Do QT Regolin I Brockerhoff W Prost W Tegude F-J High-frequency measurements on InAs nanowire field-effect transistors using coplanar waveguide contacts IEEE Trans Nanotechnol 2010 9 432 437

[B3] Datta S Dewey G Fastenau JM Hudait MK Loubychev D Liu WK Radosavljevic M Rachmady W Chau R Ultrahigh-speed 0.5 V supply voltage In0.7Ga0.3As quantum-well transistors on silicon substrate IEEE Electron Device Lett 2007 28 685 687

[B4] Ihn SG Song JI Kim YH Lee JY Ahn IH Growth of GaAs nanowires on Si substrates using a molecular beam epitaxy IEEE Trans Nanotechnol 2007 6 384 389

[B5] Soci C Bao XY Aplin DPR Wang D A systematic study on the growth of GaAs nanowires by metal-organic chemical vapor deposition Nano Lett 2008 8 4275 4282 10.1021/nl801986r 19367965

[B6] Fang SF Adomi K Iyer S Morkoç H Zabel H Choi C Otsuka N Gallium arsenide and other compound semiconductors on silicon J Appl Phys 1990 68 R31 R58 10.1063/1.346284

[B7] Yonezu H Control of structural defects in group III–V–N alloys grown on Si Semicond Sci Technol 2002 17 762 768 10.1088/0268-1242/17/8/304

[B8] Bolkhovityanov YB Pchelyakov OP GaAs epitaxy on Si substrates: modern status of research and engineering Phys Usp 2008 51 437 456 10.1070/PU2008v051n05ABEH006529

[B9] Takano Y Hisaka M Fujii N Suzuki K Kuwahara K Fuke S Reduction of threading dislocations by InGaAs interlayer in GaAs layers grown on Si substrates Appl Phys Lett 1998 73 2917 2919 10.1063/1.122629

[B10] Hayafuji N Miyashita M Nishimura T Kadoiwa K Kumabe H Murotani T Effect of employing positions of thermal cyclic annealing and strained-layer superlattice on defect reduction in GaAs-on-Si Jpn J Appl Phys 1990 29 2371 2375 10.1143/JJAP.29.2371

[B11] Cho N-H De Cooman BC Carter CB Fletcher R Wanger DK Antiphase boundaries in GaAs Appl Phys Lett 1985 47 879 881 10.1063/1.95963

[B12] Wang G Leys MR Loo R Richard O Bender H Waldron N Brammertz G Dekoster J Wang W Seefeldt M Caymax M Heyns MM Selective area growth of high quality InP on Si (001) substrates Appl Phys Lett 2010 97 121913 1 3

[B13] Morales FM García R Molina SI Aouni A Postigo PA Fonstad CG Microstructure improvements of InP on GaAs (001) grown by molecular beam epitaxy by in situ hydrogenation and postgrowth annealing Appl Phys Lett 2009 94 041919 1 3

[B14] Usui H Yasuda H Mori H Morphology and lattice coherency in GaAs nanocrystals grown on Si(001) surface Appl Phys Lett 2006 89 173127 1 3

[B15] Ishida K Akiyama M Nidhi S Misfit and treading dislocations in GaAs layers grown on Si substrates by MOCVD Jpn J Appl Phys 1987 26 L163 L165 10.1143/JJAP.26.L163

[B16] Otsuka N Choi C Nakamura Y Nagakura S Fischer R Peng CK Morkoç H High resolution electron microscopy of misfit dislocations in the GaAs/Si epitaxial interface Appl Phys Lett 1986 49 277 279 10.1063/1.97140

[B17] Sieg RM Ringel SA Ting SM Fitzgerald EA Sacks RN Anti-phase domain-free growth of GaAs on offcut (001) Ge wafer by molecular beam epitaxy with suppressed Ge outdiffusion J Electon Mater 1998 27 900 907 10.1007/s11664-998-0116-1

[B18] Wang G Loo R Simoen E Souriau L Caymax M Heyns MM Blanpain B A model of threading dislocation density in strain-relaxed Ge and GaAs epitaxial films on Si (100) Appl Phys Lett 2009 94 102115 1 3

[B19] Yamaguchi M Dislocation density reduction in heteroepitaxial III–V compound films on Si substrates for optical devices J Mater Res 1991 6 376 384 10.1557/JMR.1991.0376

[B20] Luryi S Suhir E New approach to the high quality epitaxial growth of lattice-mismatched materials Appl Phys Lett 1986 49 140 142 10.1063/1.97204

[B21] Huang FY Theory of strain relaxation for epitaxial layers grown on substrate of finite dimension Phys Rev Lett 2000 85 784 787 10.1103/PhysRevLett.85.784 10991398

[B22] Fischer A Richter H Elastic misfit stress relaxation in heteroepitaxial SiGe/Ge mesa structures Appl Phys Lett 1992 61 2656 2658 10.1063/1.108099

[B23] Zubia D Hersee SD Nanoheteroepitaxy: the application of nanostructuring and substrate compliance to the heteroepitaxy of mismatched semiconductor materials J Appl Phys 1999 85 6492 6496 10.1063/1.370153

[B24] Jain SC Willander M Maes H Stresses and strains in epilayers, stripes and quantum structures of III–V compound semiconductors Semicond Sci Technol 1996 11 641 671 10.1088/0268-1242/11/5/004

[B25] Park S-J Bai J Curtin M Adekore B Carroll M Lochtefeld A Defect reduction of selective Ge epitaxy in trenches on Si(001) substrates using aspect ratio trapping Appl Phys Lett 2007 90 052113 1 3

[B26] Carlin JA Ringel SA Fitzgerald A Bulsara M High-lifetime GaAs on Si using GeSi buffers and its potential for space photovoltaics Sol Energy Mater Sol Cells 2001 66 621 630 10.1016/S0927-0248(00)00250-6

[B27] Vanamu G Datye AK Dawson R Zaidi SH Growth of high-quality GaAs on Ge/Si1−xGex on nanostructured silicon substrates Appl Phys Lett 2006 88 251909 1 3

[B28] Shimizu M Enatsu M Furukawa M Mizuki T Sakurai T Dislocation-density studies in MOCVD GaAs on Si substrates J Crystl Growth 1988 93 475 480 10.1016/0022-0248(88)90569-6

[B29] Wellmann PJ Sakwe SA Oehlshläger F Hoffmann V Zeimer U Knauer A Determination of dislocation density in MOVPE grown GaN layers using KOH defect etching J Crystl Growth 2008 310 955 958 10.1016/j.jcrysgro.2007.11.064

[B30] Gao L Woo RL Liang B Pozuelo M Prikhodko S Jackson M Goel N Hudait MK Huffaker DL Goorsky MS Kodambaka S Hicks RF Self-catalyzed epitaxial growth of vertical indium phosphide nanowires on silicon Nano Lett 2009 9 2223 2228 10.1021/nl803567v 19413340

[B31] Miao G Zhang D Stages in the catalyst-free InP nanowire growth on silicon (100) by metal organic chemical vapor deposition Nano Res Lett 2012 7 321 1 6 10.1186/1556-276X-7-321 PMC3413516 22716780

